# Preliminary examination of noncoding mutations of esophageal squamous cell carcinoma in African Americans

**DOI:** 10.3389/fgene.2026.1779147

**Published:** 2026-04-13

**Authors:** Hayriye V. Erkizan, Robert Wadleigh

**Affiliations:** 1 Institute for Clinical Research, Washington, DC, United States; 2 Hematology and Medical Oncology, Veterans Affairs Medical Center, Washington, DC, United States

**Keywords:** African American, computational prediction, esophageal squamous cell carcinoma, exome sequencing (ES), noncoding variants

## Abstract

The incidence of esophageal squamous cell carcinoma is geographically heterogeneous and exhibits complex genomic features. We aimed to preliminarily analyze noncoding mutations identified through whole-exome sequencing. Agilent SureSelect XT Human All Exon V6+UTR was used to capture the exome library from 10 African American ESCC patients. After calling variants, we analyzed noncoding mutations using Variant Effect Predictor, CScape-somatic, HumanBase modules and Opencravat. Pathway enrichment analysis was performed using the SIGNOR database via the NDEx IQuery web tool. Our results identified noncoding variants in the 3′UTR, 5′UTR, splice site, and promoter regions. We also observed a nominal enrichment of germline variations in DNA damage repair genes among patients with ESCC.

## Introduction

Esophageal squamous cell carcinoma (ESCC) is highly lethal, with a 20% 5-year survival rate (Institute NC, 2025). ESCC exhibits significant variations in incidence rates worldwide. The incidence rate is at least 10 times higher in hot-spot areas such as eastern and central Asia, eastern and southern Africa, and parts of South America compared to many Western regions (Abnet et al., 2018; Morgan et al., 2022). Risk factors include tobacco and heavy alcohol use, poor diet, hot food, poor oral hygiene, socioeconomic status, rural living, and exposure to environmental and chemical hazards (Abnet et al., 2018; Wang et al., 2018; Conway et al., 2023; Ran et al., 2024; Deng et al., 2025). ESCC is genomically complex, with recurrent mutations in *TP53*, *CDKN2KA*, *CCND1*, *NOTCH1*, *FAT1*, *NFE2L2*, *KMT2D*, *ZNF750*, *MUC16,* and others (Morgan et al., 2022; The Cancer Genome Atlas Research Network, 2017; Zhang et al., 2020; Erkizan et al., 2021). Common copy-number alterations and structural variations involve *CCND1*, *TP63*, *SOX2*, *FGFR1*, and *CDKN2A* (The Cancer Genome Atlas Research Network, 2017; Zhang et al., 2020; Erkizan et al., 2021).

Noncoding regions that are recurrently affected in cancers are located within regulatory units. Recurrent somatic drivers in promoters and UTRs, like 5′ UTR of *TP53*, 3′ UTR of *NFKBIZ*, *TOB1*, *BRD4* deletions, *AKR1C* rearrangements, alter transcription binding and RNA Regulation (Dietlein et al., 2022; Pudjihartono et al., 2022; Rheinbay et al., 2020). Some noncoding mutations occur in lineage-specific regulatory regions, affecting protein expression, such as variations in enhancer regions that alter KLF5 and TP63 transcription factor expression in pancreatic cancers (Hay et al., 2025).

Another crucial regulatory region is the 3′UTR, which may affect mRNA stability by altering polyadenylation signals. For example, abnormal 3′UTR polyadenylation alters the expression of the tumor suppressor gene *HMGN2* in breast cancer (Xu et al., 2025).

Somatic changes in the ultraconserved elements of noncoding regions are also crucial in cancers by silencing tumor suppressor genes (*ARID1B*) and enhancing oncogenic proteins (RPS13), and by mutations in miRNAs, which are often located within ultraconserved noncoding elements (Bayraktar et al., 2025).

508 whole-genome sequencing of tumors of patients with ESCC in China showed that intergenic and intronic regions carried the highest mutation burden (Cui et al., 2020). The same study identified 112 lncRNAs, 225 3′UTRs, 34 5′UTRs, and 627 promoters that were significantly more frequently mutated than expected. Also seen in other cancers (Weinhold et al., 2014), recurrent mutations in the *WDR74* promoter were observed in the study. Noncoding driver mutations in the *SLC35E2* promoter region were associated with worse survival (Cui et al., 2020).

Many genetic risk variants for ESCC are specific to certain populations, although some important loci are common across groups. For instance, *PLCE1*, *CHECK2*, *and BRCA2* are prevalent across many populations; however, the African-specific *MYO1B* is a limited-population locus (Cui et al., 2020; Ko et al., 2020; Chen et al., 2023).

In our previous studies, we demonstrated that activation of the stress-response and detoxification pathways in African Americans with ESCC (Erkizan et al., 2017). We also previously reported a high somatic mutation rate in a subset of ESCC patients (Erkizan et al., 2021). Additionally, complex somatic copy number changes were observed, and various computational tools suggested mutations in *TP53, NCOR1, APC, KMT2C, CDKN1B,* and *NOTCH1* genes functioning as cancer drivers. However, variants of these genes predicted to be drivers were not common in other cancers. To further illuminate the complex nature of ESCC, particularly in African Americans, we analyzed noncoding variants obtained from previous sequencing (Erkizan et al., 2021) and explored germline variants in DNA damage repair genes.

## Materials and methods

We have reanalyzed the previously published exome sequencing results for noncoding readouts (Erkizan et al., 2021). Briefly, w somatic variants in the hole-exome sequencing was performed using the Agilent SureSelect XT Human All Exon V6+UTR kit on matched normal-tumor samples from 10 African American patients (nine males and one female) with advanced-stage ESCC, aged between 53 and 80 (Erkizan et al., 2021). All patients, except for one, reported tobacco use and alcohol consumption. We employed an analysis pipeline that included rigorous quality control (QC) and filtering as described previously (Erkizan et al., 2021), along with methods recommended in the Genome Analysis Toolkit (GATK) Best Practices (DePristo et al., 2011; Van der Auwera et al., 2013). All analyses were performed using variants aligned to the reference genome GRCh37. Somatic single-nucleotide variants (SNVs) and short insertion-deletions (InDels) called by at least two of three variant calling algorithms were filtered by a read depth of 50 x or higher. This analysis included variants with allele frequency (VAF) greater than 5% and rare variants with less than 1% minor allele frequency (MAF) in the African population. The oncogenic potential of somatic variants in the UTR regions was evaluated using CScape-somatic (Rogers et al., 2020). The variants with scores supporting high-confidence driver predictions were analyzed, and their effects were identified using Ensembl Variant Effect Predictor. HumanBase (https://hb.flatironinstitute org/) modules such as DeapSea, Sei, and ExPecto were used to predict the effect of the variants (Zhou et al., 2018; Sokolova et al., 2023; Park et al., 2021; Chen et al., 2022). The various features of these regions were further analyzed using Opencravat (Pagel et al., 2020). Pathway enrichment analysis was performed using the SIGNOR database via the NDEx IQuery web tool (Pillich et al., 2023). The variant effect prediction of germline DNA damage repair variants was performed using MutationTaster 2021 (Steinhaus et al., 2021).

The District of Columbia Veterans Affairs Medical Center Institutional Review Board (DC VAMC IRB #07077) approved this study. Written informed consent and approval for publication of results were obtained from each patient prior to the procedure.

## Results

**FIGURE 1:**
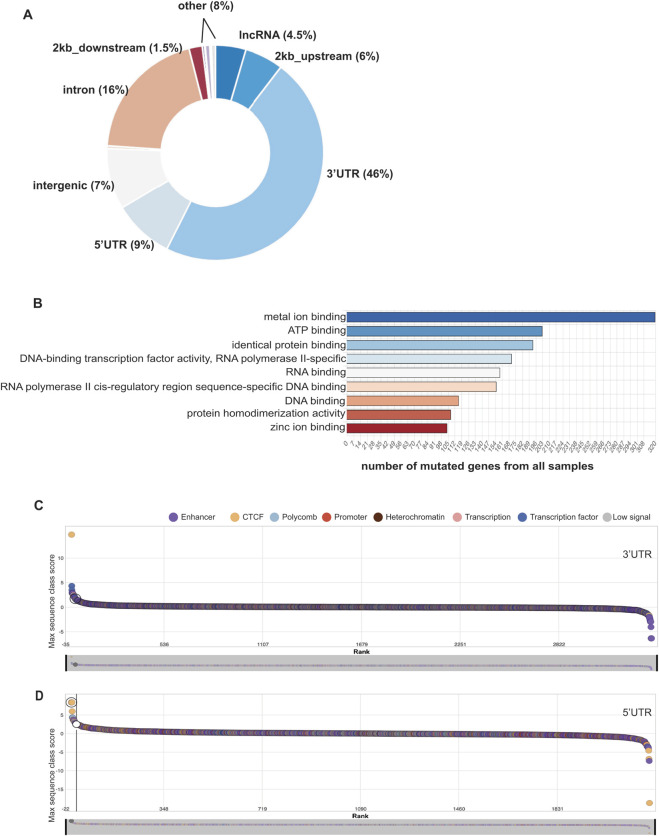
Noncoding somatic variants in ESCC. **(A)** Raw count ratio of variants in the noncoding regions. **(B)** Gene ontology analysis of the variants in the 3′UTR in ESCC. **(C)** The score rank of the 3′UTR variants predicted by the Sei module of HumanBase. **(D)** The score rank of the 5′UTR variants predicted by the Sei module of HumanBase. **(C,D)** The maximum sequence class scores are shown on the y-axis, and the class rankings are on the x-axis. The most commonly presented classes are Enhancer (purple), CCCTC-binding factor (CTCF) (orange), Polycomb (light blue), Promoter (red), Heterochromatin (dark purple), Transcription site (pink), Transcription factor binding site (dark blue), and Low signal sites that are not strongly enriched in the measured histone marks (gray). The left tail of the ranking indicates a positive prediction score, suggestion that the variant might enhance the class outcome. The right tail of the ranking indicates a negative prediction score that might have a deleterious impact on the class outcome.

In this analysis of a 10-patient cohort, we identified 5,278 noncoding variants from exome sequencing that covered 15 Mb of the noncoding genome, including UTRs, noncoding RNAs, and annotated transcripts outside of coding regions. Our previous study demonstrated that half of this cohort exhibited a high somatic coding mutation rate. Consistent with the coding mutation rate, the same samples from the high-mutation-rate group (T1, T5, T6, T7, T14) and one sample from the low-mutation-rate group (T8) total of six samples, showed an average noncoding variation rate of 92.46 ± 13.20 bp/Mb [95% CI: 79.26-105.66]. The remaining four samples (T9, T15, T16, T19) had varying amounts of noncoding variants, ranging from 6 to 58 bp/Mb. Figure 1A displays the raw count ratios of variants across different types of noncoding regions 3′ and 5′ UTRs, 2 kb upstream and downstream sequences, and, to some extent, introns with splice sites and intergenic regions.

### UTR variations

Within the captured exome regions, 3′ UTRs had the highest raw variant count (46%) (Figure 1A). The gene ontology analysis of all genes containing variations in the 3′UTR revealed an abundance of metal-ion-binding proteins, followed by ATP-binding and homodimerization protein-binding proteins (Figure 1B). Genes with variations in their 3′UTR are overrepresented in intracellular signaling and cell-cell communications, transcriptional regulation of pluripotent stem cells by OCT4, SOX2, and NANOG, RNA polymerase II transcription, RHO GTPase cycle, regulation of cytoskeletal remodeling, and transport of small molecules.

The regulatory features of 3′UTRs were analyzed using the HumanBase Sei module (Chen et al., 2022). This framework predicts 40 sequence-class scores, grouped into promoter, enhancer, CTCF-cohesin binding, transcription factor binding, polycomb-repressed, heterochromatin, transcription, and low signal sequence classes (Chen et al., 2022). The highest predicted sequence class score was 14.78, corresponding to CTCF-Cohesin at chr 1:1377151G>T (*VWA1 - von Willebrand factor A domain-containing 1* gene) (Figure 1C_left tail). Variants at chr 1:65157271 G>A and chr 1:1377151 G>T were predicted to positively impact the TF3 FOXA1/AR/ESR1 sequence class with scores of 4.32 and 3.07, respectively. The sequence class scores computed by the Sei module indicate the impact of a variant by the difference between scores for the reference and alternative alleles. Variants that score highly for a particular sequence class have high predictions for the chromatin profiles associated with that class (Chen et al., 2022). Conversely, a negative predicted score suggests that the variant may not support the class activity. Such negative sequence class scores were predicted for variants that affect the E6 weak epithelial enhancer sequence (chr 2:25168960 T>C, score of −6.36), two different E12 erythroblast-like enhancers (chr 6:14136323 T>C and chr 1:64339341 A>G, with scores of −4.06 and −2.98, respectively), and E4 multi-tissue enhancer (chr 22:41328618 G>A, score of −2.63) (Figure 1C_right tail).

Variations in the 5′UTR account for 9% of all raw noncoding variants in our dataset (Figure 1A). 5′UTR genes showed nominal enrichment in DNA repair and stress response pathways. Detailed analysis of the 5′UTRs in the Sei module of HumanBase suggested that the top three (8.41, 5.99, 3.72) and the lowest three class scores (−18.68, −6.81, −4.55) belong to CTCF-cohesion sites (Figure 1D). Some promoter sites, which also contain CTCF-cohesin, were negatively impacted with scores of −7.14, −5.72, −5.18. Various PC1 polycomb/heterochromatin and PC4 Polycomb/Bivalent stem cell enhancers in P promoter classes were also predicted to be affected. To explore this, we further used the ExPecto analysis module of HumanBase, which predicts expression levels directly from the sequence (Zhou et al., 2018). The *APOBEC3A* variant (chr 22:399353560 G>C) located in the 5′UTR was also predicted to positively influence gene expression with the Max ExPecto effect score of 0.63, where normalized scores range from 0 to 1. Molecular-level biochemical effect predictions suggested that this variant increased the probability of transcription-promoting H3K4me1 activity with a z-score of 4.7. Likewise, the variant may increase the likelihood of H3K27ac activity and decrease the probability of H3K27me3. Therefore, this *APOBEC3A* variant might alter the epigenomic landscape of APOBEC3A, leading to higher expression. *Poly(A) Polymerase Beta (PAPOLB)* has a chr 7:4901679 G>A variant, an upstream gene variant located 54 bp upstream of the transcript start site. The variant is predicted to enhance the silencing effect of H3K27me3 (z-score of 79) and reduce the activating effect of H3K4me3 (z-score of −51). As a result, the overall predicted impact of this variation on *PAPOLB* activity is negative. Likewise, the *Granzyme M (GZMM)* variant chr 19:544012 A>G and *the Apolipoprotein L6* (*APOL6*) variant chr 22:36044487 G>A are both predicted to exert a net suppressive effect on their transcription.

In our dataset, Cscape also predicted that somatic variations in the 3′UTR of 48 genes, including *ETV1*, *FOXO3*, and *MSI2*, and 7 5′UTR variants, led by *ROBO2* and *KLF4,* might exert an oncogenic effect. Any Cscape score above 0.7 indicates a prediction of potential tumorigenic function for noncoding variants shown in Table 1.

**TABLE 1:** Tumorigenic genes affected by mutations in 3′UTR and 5′UTR.

Gene	CScape_Score*	Driver_Class
3′UTR		
*ETV1*	0.842	O, F
*FOXO3*	0.833	O, F, TSG
*MSI2*	0.833	O, F
*PCM1*	0.824	F
*AFF4*	0.824	O, F
*TCF7L2*	0.822	O, F
*KAT6A*	0.821	O, F
*FOXO1*	0.814	O, F, TSG
*IRF4*	0.808	O, F, TSG
*TRIM24*	0.798	O, F, TSG
*BCL11B*	0.798	O, F, TSG
*EML4*	0.798	F
*NCOA2*	0.795	O, F
*ARHGAP5*	0.792	O
*ELK4*	0.787	O, F
*RABEP1*	0.785	F
*FGFR1*	0.785	O, F
5′UTR		
*ROBO2*	0.777	TSG
*KLF4*	0.775	O, TSG
*EP300*	0.773	F, TSG
*SETBP1*	0.715	O, F
*CACNA1D*	0.713	O
*BCL2*	0.701	O, F
*BRCA2*	0.700	TSG

* Cscape Score: CScape Score >0.7 is considered significant for noncoding variants.

O: oncogene, F: fusion, TSG: tumor suppressor genes.

### Intronic variants

Intronic variants are over-represented in pathways related to organelle biosynthesis and maintenance, oncogene-induced senescence, PI3Kgamma signaling, RHO GTPase, NOTCH1-regulated signaling, and inflammatory signaling involving IL-33 and IL-6. Among these intronic variants, ten genes are predicted to have a tumorigenic effect (Table 2).

**TABLE 2:** Tumorigenic genes affected by mutations in introns.

Gene	CScape_Score*	Driver_Class
*RAP1GDS1*	0.980	O, F
*STAT3*	0.972	O
*PRDM2*	0.807	TSG
*BRD4*	0.786	O, F
*GATA2*	0.769	O
*DCTN1*	0.748	F
*CAMTA1*	0.736	TSG, F
*SMARCE1*	0.721	TSG
*AKT1*	0.708	O
*ALDH2*	0.701	F

* Cscape Score >0.7 is considered significant for noncoding variants.

O: oncogene, F: fusion, TSG: tumor suppressor genes.

### Splice-site variants

In our analysis, we rarely observed splice site variants. Although intronic sites accounted for 16% of all raw variants, splice-site variants were infrequent in our cohort, comprising 0.87%–2.7% of all raw SNVs. Four samples had a splice-site mutation in *MAP4K5*, a serine/threonine kinase involved in epithelial-to-mesenchymal transition (Wang et al., 2016). Another four samples demonstrated a splice-site mutation in the autophagy protein-coding gene *C12orf44*, also known as *ATG101* (Nagy et al., 2014). Twenty-three splice-site mutations affecting RNA metabolism, RHO GTPases, SUMOylation, glucose metabolism, heat stress response, and mitochondrial calcium homeostasis were observed in three tumor samples. Insertions in the *CD36* gene in three samples and in the *PBRM*1 gene in two samples created splice-site variants. An SNV in 17:g.7576852C>T in *TP53* resulted in a splice site variation in two samples.

### Promoter variants

Esophageal tissue expresses nearly 70% of the total human proteins, and only 435 proteins are expressed at a high level compared to other tissues or specifically in the esophagus (Uhlen et al., 2015; Fagerber et al., 2014). These proteins function in cell-to-cell communication, gap-junction trafficking and regulation, interleukin signaling and the innate immune system, lipid metabolism, fibronectin matrix formation, transcriptional regulation by TP53, PTK6 expression, and keratinization. We observed that 205 of these esophageal genes were mutated in our samples (Supplementary Table S1). Except for interleukin signaling and lipid metabolism, all other esophageal-specific proteins lost function due to mutations. With captured promoter regions, TFAP2A binding sites accounted for 14% of observed variation sites; motif-level enrichment relative to background was not formally assessed.

In addition, 2900 variants were identified within the 20 kilobases from the nearest transcription start site. These variants were analyzed by *in silico* prediction of disease-associated regulatory variation (Zhou et al., 2018). *TSPAN13* (chr7:16793542 C>T) was predicted to have a Max ExPecto expression effect of 0.97, the highest in our cohort. Molecular-level biochemical effect predictions included a z-score of −18 at the gene silencing mark H3K27me3. A negative z-score indicates that a mutation decreases the probability of a regulatory feature, therefore, predicting increased expression of tetraspanin 13 (TSPAN13). This protein has a role in suppressing CD8^+^ T-cell infiltrations, as shown in breast cancer cells (Yin et al., 2026). However, the immune evasion role of TSPAN13 has not been studied in esophageal cancer cells.

### Germline variants

**FIGURE 2:**
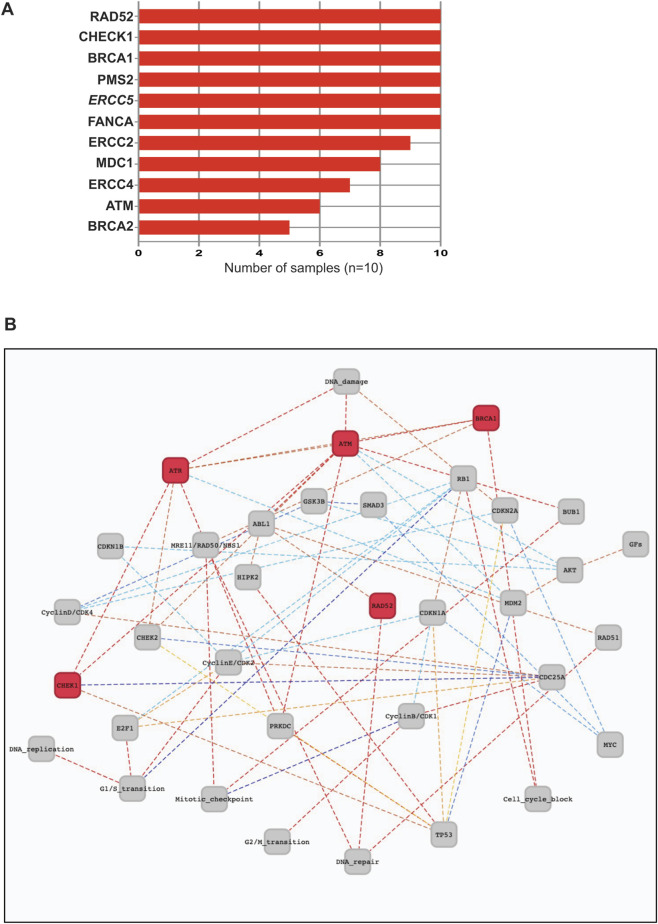
Germline variants of DNA Damage Repair genes in ESCC. **(A)** Histogram of germline variants of DDR genes in 10 ESCC samples. **(B)** NDEx Signor Pathway Enrichment summary of DDR genes.

Recent studies have shown that the most common group of mutated cancer susceptibility genes is vital to DNA damage repair (DDR) and that there is a high prevalence of germline DDR genes in patients with advanced and metastatic cancers (Sailer et al., 2019). We observed diverse germline variants within 21 DDR genes in this cohort (Supplementary Table S2). Ten of those genes were found in five or more normal tissue samples (Figure 2A). Genes *RAD52*, *CHECK1*, *ATM*, *MBN*, *ATR*, and *BRCA2* function in the G2/M phase transition pathway (Figure 2B).

The highest Combined Annotation-Dependent Depletion (CADD) score of 34 corresponded to the amino acid substitution P493L in the Bloom Syndrome RexQ helicase (BLM) protein, as observed in two normal tissues from two patients.

Rare variants in *PARP1, ERCC5, MDC1, ERCC2, POLE, ATM, BRCA1,* and *FANCA* genes, as well as in noncoding regulatory regions and noncoding RNA genes, might have deleterious functional effects (Supplementary Table S2). MutationTaster predicted deleterious effects of *ERCC2* with two different variants (chr19:45855524 C>T (D633E or D711E) and chr19:45867259 G>A (D288Y) and *PARP1* chr1:226555302 T>C leading to V762G in all 10 control samples.

Five distinct germline SNPs in *TP53* (rs2909430, rs1042522, rs59758982, rs1642785, rs8073498) were detected in control tissues for all 10 of our patients. The SNP, rs1042522 (NC_000017.10:g.7579472 G>C), leads to the missense mutation Pro72Arg, whereas the other variants are located in noncoding regions of *TP53*.

## Discussion

In this study, we explored the noncoding variants as a complement to our previously published coding mutation analysis (Erkizan et al., 2021). These samples exhibited a total of 5378 genomic noncoding somatic variants. We determined that the noncoding variation rate aligned with the somatic coding mutation rate for each tumor sample in our cohort, except for one. This trend is expected, since more than 98% of the genome is noncoding; the mutation rate obtained from exome sequencing is representative of the whole genome. In pan-cancer whole-genome sequencing cohorts, average mutation rates range from 0.01 mutations/Mb in low-mutation pediatric tumors to over 20 mutations/Mb in highly mutated tumors (Lochovsky et al., 2015). Tumors in our cohort had relatively higher somatic variation rate; however, given the exome sequencing and the heterogeneous nature of sequencing coverage, direct comparison of the mutation rate of ESCC in the literature may not be appropriate. Most noncoding variations are considered passengers. But recurrent hotspots, especially in promoter, enhancer, UTR, and noncoding RNA sequences, might have tumorigenic consequences (Dietlein et al., 2022; Rheinbay et al., 2020). In our analysis, we found that almost half of the raw somatic noncoding variants occurred mainly in the 3′UTRs of genes. As previously shown, a widespread, recurrent shortening of the 3′UTR in ESCC may drive tumor progression (Lin et al., 2021). However, in our dataset, we observed a high number of variations rather than length changes of the 3′UTR. The Sei module analysis of 3′UTR showed enrichment of variants in transcription factors FOXA1, AR, and ESR1, grouped as TF3 in the Sei module. These transcription factors are tightly coupled, hormone-dependent transcriptional network in epithelial cells (Bernar et al., 2012; Zhao et al., 2016). FOXA1 binds compact chromatin and opens nucleosomes, thus creating accessible enhancers and facilitating AR and ESR1 binding to their response elements (Chaudhary et al., 2017; Jin et al., 2014; Yang and Yu, 2015). As previously shown, high Androgen Receptor (AR) expression correlates with shorter overall and disease-free survival in ESCC (Chaudhary et al., 2017; Dong et al., 2017). Mechanistic studies suggested that AR might drive invasion and metastasis, and provide sustained inflammation and signaling crosstalk with IL6 and STAT3 (Dong et al., 2017; Cai et al., 2023). Alterations in miRNA binding sites within the 3′UTR can affect gene expression, as seen with FOXJ3 overexpression and CLOCK downregulation in ESCC (Chang et al., 2017). In our dataset, somatic variants in the 3′UTR overrepresented transcriptional regulation of pluripotent stem cells by OCT4, SOX2, and NANOG, RNA polymerase II transcription, the RHO GTPase cycle, and regulation of cytoskeletal remodeling, resulting in aggressive, stem-like, highly plastic, and invasive states, which is concurrent with the advanced stage of our samples. Deeper analysis of these sequences supported the loss of tissue-specific regulatory signatures through functional switches in tissue-specific gain-of-function in multi-tissue and epithelial enhancers.

Somatic 5′UTR variants impacted genes in DNA repair pathways, including alternative non-homologous end-joining repair and ATM-mediated repair. These pathways are considered promising targets for cancer therapy. However, large-scale whole-genome datasets reveal many recurrently mutated 5′UTRs, but the specific genes we predicted are not identified as drivers (Cui et al., 2020).

Further analysis of our cohort using deep-learning-based modules predicted that variants in 5′UTR sites were enriched for variants in CTCF-Cohesin sequences. Folding of genomic DNA into loops is coordinated by cohesion, an essential factor in chromatin organization and gene regulation. The DNA-binding protein CCCTC-binding factor (CTCF) controls loop extrusion and three-dimensional genome architecture (Davidson et al., 2023). CTCF acts as a transcriptional regulator and insulator, blocking enhancer-promoter communication, so CTCF has both cis- and long-range genome interaction effects (Chen et al., 2022; Fang et al., 2020). A previous study demonstrated an exceptionally high mutation rate (3.31-fold) at functional CTCF-binding sites in various cancers compared to control sites (Kaiser et al., 2016). The most frequently observed substitution at CTCF-binding sites is T-to-G/C/A, the strongest T-to-C signature (Kaiser et al., 2016). Mutational signature analysis of this cohort in our previous publication also showed an excess of the T-to-C signature, indicating that the SBS16 signature dominates in high-mutation-rate samples (Erkizan et al., 2021). Underlying mutational events may cause genome-wide trends, and CTCF binding sites follow these mutational biases. The functional CTCF motifs were found to be prone to high-frequency mutations when located at chromatin loop anchor points and domain boundaries (Kaiser et al., 2016). Therefore, the CTCF sites involved in higher-order chromatin structures may bear the highest mutational burden, thereby strikingly affecting chromatin organization (Kaiser et al., 2016).

Additional evolutionary analysis based on human population variant allele frequencies indicates that variants within CTCF sequence classes have lower common-variant allele frequencies and exhibit stronger overall negative selection constraints (Chen et al., 2022). In ESCC, altered methylation in regulatory regions, including CTCF binding sites, is associated with cancer-specific transcriptional changes. Hypermethylation of specific CTCF-binding sites in promoters, such as the *WNT2* promoter, increases WNT2 expression and drives invasion and metastasis in ESCC (Cao et al., 2020). Even though this study emphasizes the functional role of CTCF binding sites in ESCC, the role of CTCF binding site mutations in esophageal squamous cell carcinoma has not been explored in the literature.

In our study, the lowest sequence class score for the CTCF-cohesin site occurred in the 5′UTR of *PRPF38A*, a pre-mRNA processing factor. PRPF38A acts in RNA splicing and is associated with the U4/U6.U5 tri-snRNP complex (Chan et al., 2017). PRPF38A was recently suggested as a late-outcome marker in colorectal cancer (Yu et al., 2025). Further studies are needed to elucidate the mutation burden of CTCF binding sites and functional consequences in ESCC.

We previously reported a high somatic mutation rate in this patient cohort (Erkizan et al., 2021). The median mutation rate was greater than that of most tumors, ranked between cutaneous melanoma and lung squamous carcinoma in TCGA panel (Erkizan et al., 2021). In high-mutation-rate cases, we detected the SBS26 mutational signature at approximately 15% and found it to be closely correlated with defective DNA mismatch repair and microsatellite instability (Erkizan et al., 2021). Therefore, we sought to identify possible reasons for the high somatic mutation rate. Germline DDR gene defects, particularly in those that proofread and ensure the replicative fidelity of DNA, such as *POLE*, may increase tumor mutation burden (Ying et al., 2021).

Although we observed three distinct *POLE* variants, none were predicted to have deleterious effects. Among the other 20 DDR genes, *BLM*, which encodes a DNA helicase reported to stabilize the genome, harbored a missense mutation with a high deleteriousness score (CADD) in 2 control tissues. *BLM* haploinsufficiency can be oncogenic, with low to moderate penetrance in early-onset colorectal cancer risk (de Voer et al., 2015). However, there is no direct evidence linking BLM protein functions to ESCC biology.

Additionally, germline variants were displayed in 21 DNA damage repair genes. *RAD52*, a gene involved in the repair of DNA double-stranded breaks, carried greater than 20 distinct variations in 10 control tissues in our cohort. This *RAD52* locus confers genetic susceptibility to squamous cell cancers of the upper aerodigestive tract (Delahaye-Sourdeix et al., 2015). Germline variants in DNA Damage Repair genes may modulate cancer risk, as studied in non-small cell carcinoma (Zienolddiny et al., 2006). In that study, the *ERCC2* gene with variants leading to K751Q was identified as one of the DDR genes that modulate cancer risk.

However, most germline DDR variants identified in large datasets remain variants of uncertain significance. Therefore, further functional assays are necessary to clarify their potential cancer risks. Furthermore, germline DDR variants may influence treatment sensitivity in head and neck cancers. Ten variants in *TP53, ATM*, *XRCC1*, and *XRCC3*-related loci could predict failure of chemo-radiotherapy and poor survival (Butkiewicz et al., 2023).

The mutational event in the *TP53* gene, unlike other ESCC cohorts, was limited to a splice site mutation to lead to splice site loss (c.516 + 1G>A) in two patients. Previously, this rarely seen splice-site variation has been described in various cancers, including head and neck squamous cell carcinoma and ESCC (Cheng et al., 2016; Wong et al., 2018; Temam et al., 2000). In ESCC, truncating *TP53* mutations were observed in the Pro72 allele but not in tongue squamous cell carcinoma, suggesting tissue-specific biology (Adduri et al., 2014).

All of the *BRCA1* and *BRCA2* variants identified in our results are considered common polymorphisms, not pathogenic, and may not increase the potential cancer risk. Conversely, the *BRCA1* p. P871L variant is associated with a decreased overall cancer risk, particularly in the Asian population (Miao et al., 2017).

Our analysis was limited to DNA sequencing only due to the size of the biological samples and the technology available at the time. Therefore, the effect of the promoter variants could have been easily tested by RNA sequencing, but our analysis was confined to DNA.

Given the small sample size (n = 10), the advanced-stage composition, and the exposure profile, these results should be interpreted as cohort-specific and exploratory.

Future studies are needed to verify the significance of noncoding gene variations in a larger cohort and to delineate their functional roles in ESCC.

## Data Availability

The original contributions presented in the study are included in the article/Supplementary Material, further inquiries can be directed to the corresponding author.
